# Role of Homer Proteins in the Maintenance of Sleep-Wake States

**DOI:** 10.1371/journal.pone.0035174

**Published:** 2012-04-20

**Authors:** Nirinjini Naidoo, Megan Ferber, Raymond J. Galante, Blake McShane, Jia Hua Hu, John Zimmerman, Greg Maislin, Jacqui Cater, Abraham Wyner, Paul Worley, Allan I. Pack

**Affiliations:** 1 Center for Sleep and Circadian Neurobiology, University of Pennsylvania, Philadelphia, Pennsylvania, United States of America; 2 Division of Sleep Medicine, Perelman School of Medicine, University of Pennsylvania, Philadelphia, Pennsylvania, United States of America; 3 Kellogg School of Management, Northwestern University, Chicago, Illinois, United States of America; 4 Department of Neuroscience, Johns Hopkins University, Baltimore, Maryland, United States of America; 5 The Wharton School, University of Pennsylvania, Philadelphia, Pennsylvania, United States of America; Simon Fraser University, Canada

## Abstract

Sleep is an evolutionarily conserved process that is linked to diurnal cycles and normal daytime wakefulness. Healthy sleep and wakefulness are integral to a healthy lifestyle; this occurs when an organism is able to maintain long bouts of both sleep and wake. Homer proteins, which function as adaptors for group 1 metabotropic glutamate receptors, have been implicated in genetic studies of sleep in both *Drosophila* and mouse. *Drosophila* express a single Homer gene product that is upregulated during sleep. By contrast, vertebrates express Homer as both constitutive and immediate early gene (H1a) forms, and H1a is up-regulated during wakefulness. Genetic deletion of Homer in *Drosophila* results in fragmented sleep and in failure to sustain long bouts of sleep, even under increased sleep drive. However, deletion of Homer1a in mouse results in failure to sustain long bouts of wakefulness. Further evidence for the role of Homer1a in the maintenance of wake comes from the CREB alpha delta mutant mouse, which displays a reduced wake phenotype similar to the Homer1a knockout and fails to up-regulate Homer1a upon sleep loss. Homer1a is a gene whose expression is induced by CREB. Sustained behaviors of the sleep/wake cycle are created by molecular pathways that are distinct from those for arousal or short bouts, and implicate an evolutionarily-conserved role for Homer in sustaining these behaviors.

## Introduction

Sleep is essential for all organisms that exhibit diurnal attention. A current model of sleep/wake control proposes that sleep and wake are created by a “flip-flop” involving reciprocal inhibition between sleep-active versus wake active neural pathways that project broadly to the neocortex and subcortical areas [1]. Neuronal groups that are active in sleep are largely limited to the ventro-lateral preoptic area [1]. Pathways that are wake active include noradrenaline cells in the locus coeruleus [2], histamine cells in the tuberomammillary nucleus [3], dopamine cells in the periaquaductal gray [4] and orexin cells in the lateral hypothalamus [5]. In support of the flip-flop model, optogenetic activation of orexin cells is sufficient to wake animals from sleep [6].

While this model has been successful, it is not sufficient to explain sleep/wake control. First, targeted lesions of wake/active neuronal groups that would be expected to change the amounts of sleep and wake do not do so [7]. Second, in mice, both sleep and wake exist in two forms–short bouts, which are relatively frequent, and long, sustained or consolidated bouts. In healthy rodents and humans, consolidated sleep occurs after the onset of the inactive period while consolidated wakefulness occurs shortly after the beginning of the active period. Consolidated sleep is important as it enhances memory and learning [8]. Here, we report a role for Homer in sleep/wake behavior, in particular consolidation of state. This role would not be predicted by the flip-flop model and hence a new mechanism to consolidate behavioral state that involves Homer is described.

Homer proteins function as molecular adaptors that bind to a specific proline-rich sequence in the C-terminus of Group I metabotropic glutamate receptors and other proteins that play a role in Ca^2+^ signaling. The vertebrate genome includes three Homer genes (Homer 1, 2, 3). Conventional Homer proteins are bipartite, consisting of an N-terminal Enabled/Vasp homology 1 (EVH1) target-binding domain and a C-terminal coiled-coil (CC) domain that mediates self-association. Homer1a and Ania 3 are immediate early gene (IEG) forms that lack the CC domain and function as dominant negatives to disrupt Homer cross-linking [9], [10]. Thus, up-regulation of H1a is functionally equivalent to down regulation of the cross-linking Homer molecule. Drosophila possess a single *homer* gene that encodes a cross-linking Homer protein but no Homer-1a homologue [11]. That Homer might be involved in sleep/wake control is suggested by three observations: 1) Homer in Drosophila is up-regulated during sleep and down-regulated during extended wakefulness [12]; 2) in mice, the dominant negative Homer1a is up-regulated during wakefulness and down-regulated during sleep [13], [14], ; and 3) variation in Homer1a regulation is a strong candidate to explain the differential response of different inbred mouse strains to sleep loss [15], [17]. Despite these intriguing associations between Homer and sleep/wake control, it remained unclear whether changes in expression play a causal role in sleep/wake control or are simply a correlate of these behavioral states. To address this issue we performed a comparative analysis of sleep/wake behavior using Drosophila and mouse genetic models. Drosophila lacking Homer sleep less and have markedly shorter bouts of sleep. Mice lacking Homer1a show reduced wakefulness with an inability to sustain long bouts of wakefulness in the normally active period in the lights off part of the day. H1a KO mice also display a modest change in circadian period. These findings indicate that sustained behaviors of sleep and wake share an evolutionarily conserved dependence on Homer, with slightly different mechanisms because the Homer molecular repertoire has become more complex in mammalian species.

## Results

### Drosophila Homer protein levels change with sleep deprivation

The *homer* transcript in adult female Drosophila brains is down regulated with sleep deprivation and increased with sleep [12]. Accordingly, we examined Homer protein levels in heads of adult female of the white Canton-S 10 (CS) strain that had been sleep deprived for 6 hours, between ZT16–22. Homer protein was detected in the heads and was reduced by ∼40% in flies not allowed to sleep compared to undisturbed flies that were sacrificed at the same diurnal time (p = 0.044) (Figure S1).

### Sleep is reduced and fragmented in the Homer null flies

**Figure 1:**
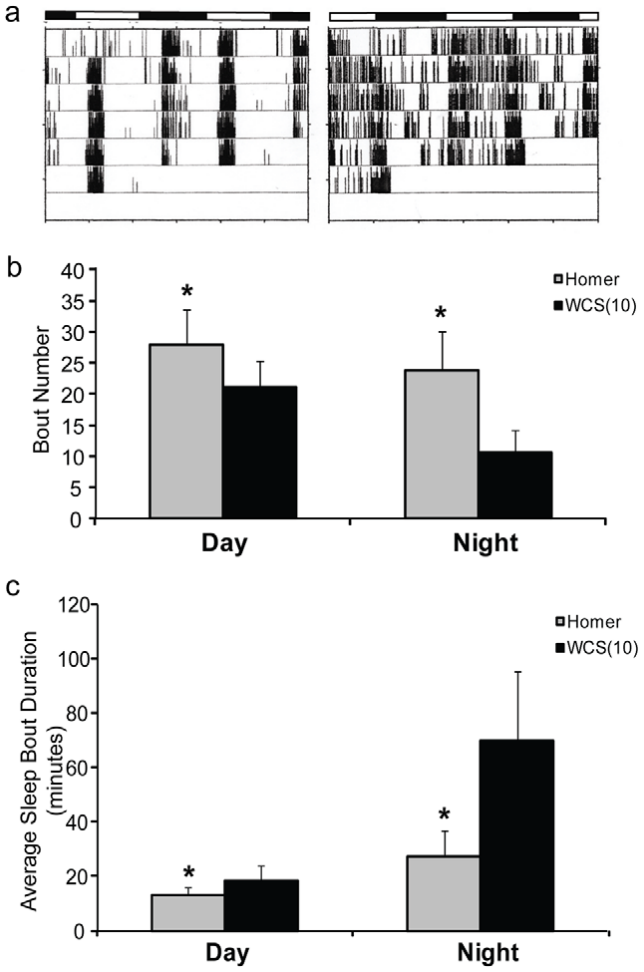
Loss of Drosophila Homer alters sleep architecture. A) Representative actograms displaying activity in *homer* ^R102^ (right panel) and CS (left panel) flies under a 12∶12 L:D regimen. Bar at the top denotes light and dark periods. B) The average number of sleep bouts and standard deviation during the day and night in Homer^R102^ (gray bar; n = 47) and the wildtype CS flies (black bar; n = 65) as determined by video analysis. The average number of sleep bouts are significantly greater in homer flies (*p<0.0001). C) Histogram showing the average sleep bout duration and standard deviation during the day and night in Homer^R102^ (gray bar; n = 47) and the wildtype CS flies (black bar; n = 65) as determined by video analysis. The average sleep bout is significantly shorter in homer flies (*p<0.0001). This difference is particularly marked at night.

**Figure 2:**
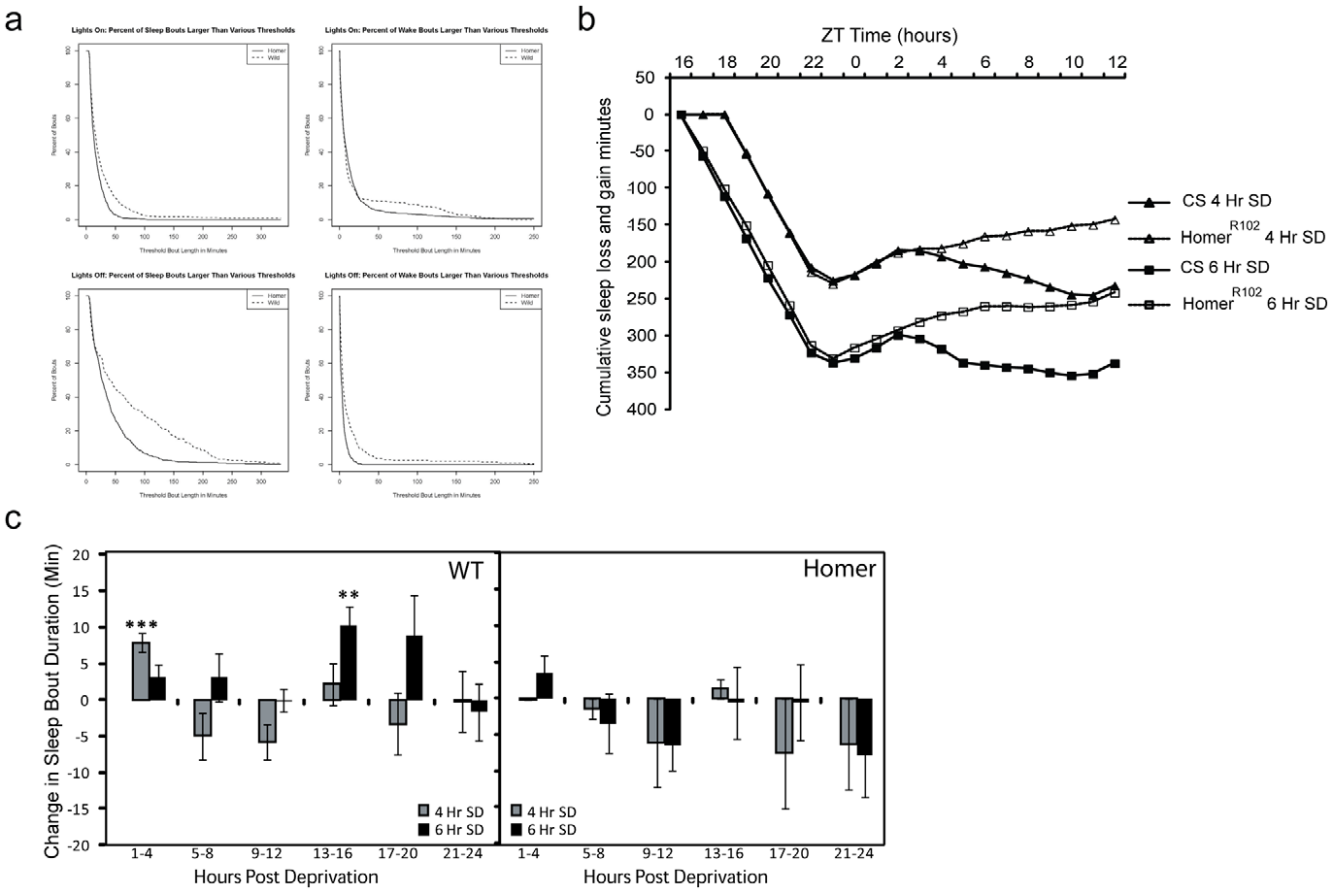
The response to sleep deprivation is altered in Homer null flies. A) Distribution of sleep and wake bouts during the lights on (top panels) and lights off (bottom panels) periods in Homer^R102^ and wildtype flies. Sleep was assessed using 5 second epochs. Both sleep and wake bouts are shorter in the Homer mutant flies. The most striking difference occurs during the night where the average sleep bouts are much shorter in the homer mutant flies than in the background strain. B) Recovery sleep profile following 4 and 6 hr SD in homer^R102^ and CS flies. Plot illustrates the cumulative sleep loss and gain over 12 hours following sleep deprivation. Rebound recovery sleep lasts longer in homer^R102^ than in wildtype flies. C) Effect of sleep deprivation upon sleep bout duration. Panel A: wildtype flies: the average delta in bout duration ± standard deviation post sleep deprivation is shown for 4 and 6 hour SD. Sleep bout duration lengthens after SD for wildtype flies deprived for 4 and 6 hours. Panel B: Homer null flies do not show any lengthening of sleep bouts when sleep deprived for 4 or 6 hours. Bout duration changes which are significantly different from baseline are designated as follows: *<0.05>0.01; **<0.01>0.001, ***<0.001.

We assessed sleep-wake activity in *homer^R102^* mutant flies (n = 120) by electronic beam breaks (Drosophila Activity Monitoring System, DAMS) under 12∶12 light-dark conditions and compared the activity pattern to that of the CS (n = 102) background strain. As DAMS analysis can overestimate the amount of daytime sleep and bout durations of state, both during the day and at night, we also used video recording under the same conditions [18] (Table 1). By DAMS, Homer mutant flies display fragmentation of sleep compared with the CS flies as is illustrated by the locomotor actograms in Figure 1a. Homer^R102^ flies have less sleep compared to controls both by DAMS and video (p<0.0001). The average bout duration of sleep is much reduced in the Homer^R102^ compared to wildtype flies during the lights on (p<0.0001) and lights off (p<0.001) periods (Figure 1c and Table 1). By video we found that the amount of sleep was not significantly different during the day but was reduced at night in the Homer^R102^flies (p<0.001) (Table 1). This difference in sleep is related to the inability of the fly to sustain sleep since the flies display more and shorter sleep bouts (Figure 1b and 1c and Table 1). Using 5 second epochs we examined the distribution of bouts of different durations of inactivity and we find that the loss of function of Homer flies have much shorter bouts of quiescence with reduced long bouts of sleep compared to WT flies (Figure 2a). This effect is more pronounced during the lights off period when flies “sleep”. There are also effects on wake at night but these are minor. The average sleep bout duration at night in Homer null flies is more than 50% shorter than that in the CS background strain. Wake bout durations are also significantly shorter in the Homer^R102^ compared to wildtype both during the day and at night (Table 1). The differences are, however, not as marked as the difference in sleep bout duration at night.

**Table 1:** Sleep-wake data from homer null and WCS(10) flies over 24 hr and during the day and night using video analysis.

	WCS(10)(n = 65)	Homer^R102^(n = 47)	p-value
Minutes Total Sleep/24hr	992.24±64.79	928.59±129.70	<0.001
Minutes Active/24hrs	447.73±64.78	511.37±129.70	<0.001
Sleep Bout Number	30.0±7.3	48.0±10.6	<0.0001
Wake Bout Number	30.1±7.4	47.5±10.7	<0.0001
Avg Sleep Bout Duration (min)	36.2±9.4	23.3±6.9	<0.0001
Avg Wake Bout Duration (min)	14.3±3.9	9.1±3.4	<0.0001

Data for Lights on Period			
Minutes Total Sleep	363.98±56.43	365.94±98.96	NS
Minutes Active	355.99±56.43	354.04±98.96	NS
Sleep Bout Number	21.12±4.21	27.98±5.71	<0.0001
Wake Bout Number	21.77±4.21	28.46±5.62	<0.0001
Avg Sleep Bout Duration (min)	18.46±5.65	13.14±2.85	<0.0001
Avg Wake Bout Duration (min)	17.33±4.23	14.30±7.57	0.008

Data for Lights Off Period			
Minutes Total Sleep	628.0±27.0	562.0±47.0	<0.0001
Minutes Active	91.73±27.53	157.33±47.92	<0.001
Sleep Bout Number	10.68±3.58	23.85±6.24	<0.0001
Wake Bout Number	11.09±3.58	24.07±6.24	<0.0001
Avg Sleep Bout Duration (min)	69.54±25.88	27.04±9.83	<0.0001
Avg Wake Bout Duration (min)	8.95±3.22	6.90±2.05	<0.001

### The sleep phenotype maps to the *homer* gene

To confirm that the sleep phenotype we observed in Homer^R102^ mutant flies is due to deletion of the *homer* gene, we crossed the Homer^R102^ flies with a deficiency line Df(2L)ED7007 which lacks a small region of the left arm of chromosome 2 (bands 27A1–27C7) that includes *homer* (located on 27A1). The *homer* **/**Df (2L) ED7007 hemizygotes (n = 10) displayed sleep behavior similar to the *homer* homozygotes (n = 16). In all sleep wake parameters *homer^R102^/Df(2L)ED7007* is not significantly different from *homer^R102^/homer^R102^* but is significantly different from CS/*Df(2L)ED7007* (p<0.001 for number of sleep bouts, mean bout length) and *homer^R102^*/*CS* (p = 0.0001 for number of sleep bouts, mean bout length, p = 0.001 for total sleep) (Figure S2). Expression of *Homer* in the Homer^R102^ flies using the Gal4-UAS system will definitively demonstrate the requirement of Homer for sleep consolidation; these studies are currently in progress.

### The homer^R102^ mutant flies have a normal circadian period

Examination of the circadian clock of Homer^R102^ flies (n = 16) indicate that they are rhythmic under conditions of constant darkness. No significant difference was found in clock period between the Homer null (23.6±0.2 hr; mean±SD) and the wildtype CS (23.8±0.1 hr).

### Homer^R102^ flies are not able to maintain long sleep bouts even under increased sleep drive

Wildtype and Homer null mutants show recovery sleep immediately following the end of sleep deprivation for each duration of deprivation (4, 6 ours). The immediate response in the first 4 hours after the end of deprivation was similar between genotypes. However, in *homer^R102^* flies, there was continued rebound sleep over the entire 24 hours after the end of deprivation, while in wildtype CS flies the recovery was relatively short (Figure 2b) There was a significant genotype by sleep deprivation interaction (p<0.004 for all durations of deprivation). Thus, both the CS background strain and *homer^R102^* flies display rebound sleep following sleep deprivation but the pattern of recovery is different.

Another behavioral response to sleep deprivation is an increase in sleep bout duration [19]. Sleep deprived CS flies display a significant increase in average sleep bout duration following 4 and 6 hours of sleep deprivation (Figure 2c). In contrast, Homer^R102^ flies showed no increase in average sleep bout duration following 4 or 6 hours of sleep deprivation, but rather, increase their sleep by increasing the number of sleep bouts. Thus, Homer^R102^ flies are not able to maintain long sleep bouts even under increased sleep drive.

### Homer1a knockout mice have reduced wakefulness during the active period

**Figure 3:**
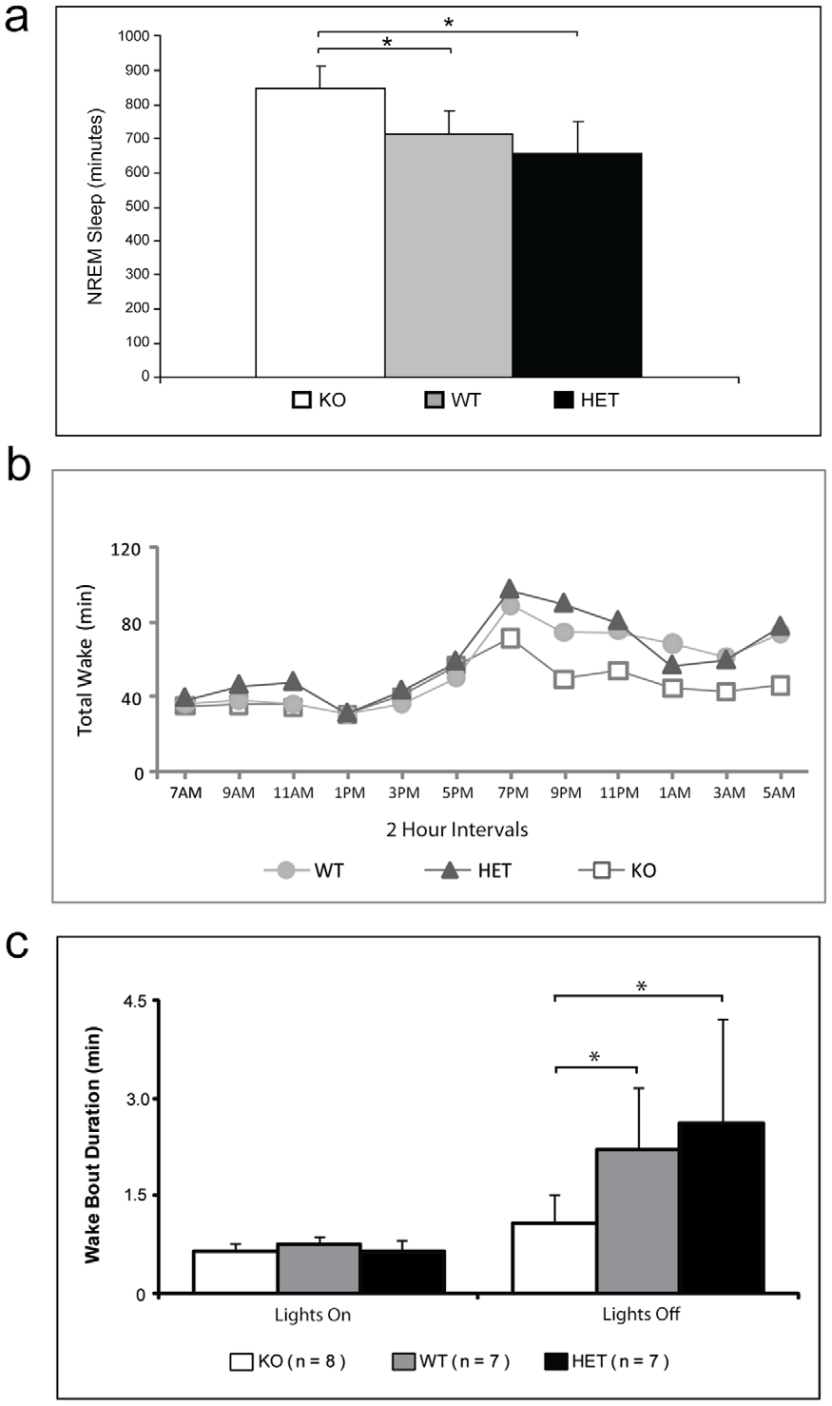
Loss of Homer1a reduces wakefulness. A) Total minutes of NREM sleep (left panel) and wake (right panel) over 24 hours in Homer1a knockout, Homer1a heterozygous and wildtype littermate mice. Homer1a KO mice have significantly more NREM sleep compared to the WT [847.3±65.4 minutes vs. 711.7±71.5 minutes (p = 0.006)] or Homer1a HETs (655.2±93.5.4 minutes [p = 0.0004)]. B) Total waking in all 3 genotypes across the 24 hour baseline day; averaged data are shown in 12×2 hour time periods. Homer1a KO exhibits less waking than either wildtype or heterozygotes but only during the nighttime active period. C) The average length of wake bouts is significantly reduced during the lights out but not the lights on period in the Homer1a knockout (KO) compared to the Homer1a heterozygotes (HET; p = 0.04) and wildtype (WT; p = 0.01) littermates. Mean wake bout duration and standard deviation are shown.

We used Homer1a KO, Homer1a heterozygous and wildtype littermate controls as the best available mouse model to study the role of Homer1a in sleep/wake behavior. H1a KO mice show a selective loss of IEG forms of Homer without changes in Homer 1c expression or expression of other synaptic proteins, and are healthy, fertile and robust [20]. The H1a KO model was selected in preference to Homer 1 KO since it is the expression of Homer 1a that is altered between sleep and wakefulness [13], [14], [15], [16] and Homer1 KO mice have no alteration in sleep/wake behavior [15]. We analyzed sleep and wake behavior over 24 hours using a one-way ANOVA, with the main factor being genotype. For non-rapid eye movement (NREM) sleep there was an overall main effect of genotype over 24 hours (F[2], [19] = 12.4; p = 0.004). Homer1a KO mice had significantly more NREM sleep compared to the wildtype littermates [847.3±65.4 minutes vs. 711.7±71.5 minutes (p = 0.006)] or Homer1a heterozygotes (655.2±93.5.4 minutes [p = 0.0004)] (Figure 3a). There was no significant difference in amount of NREM sleep between the Homer1a heterozygotes and the wildtype littermates. The difference among genotypes for rapid eye movement (REM) sleep was not statistically significant across 24 hours.

Analysis of the wake data over 24 hours indicated an overall main effect of genotype (F[2], [19] = 14.4; p = 0.0002). Homer1a KO mice display less wakefulness (541.2±64.9 min) than either the Homer1a heterozygotes (740.1±88.3 min; p = 0.0001) or wildtype littermates (672.6±65.6 min; p = 0.005). The heterozygotes were not significantly different from the wildtype littermate control mice.

Further analysis of the sleep/wake data was carried out on two 12 hour periods to examine day and night effects on genotype (Table 2). The increased NREM sleep for the Homer1a KO compared to both the heterozygous and wildtype littermate control mice occurred only during the active dark period. Increased REM sleep for the Homer1a KO (25.32±6.46 min) compared to both the heterozygous (14.65±4.98; p<0.05) and wildtype (16.59±6.63; p<0.05) littermate control mice also occurred during the active dark period.

**Table 2:** Comparison of sleep wake data in Homer1a knockout, Homer1a heterozygote and wildtype littermate mice during the lights off and lights on periods.

	Homer1a wildtype	Homer1a HET	Homer1a KO
Lights on			
Wake (min)	228.58±29.02	272.82±48.8	232.45±49.66
Wake Bout Duration (min)	0.75±0.14	0.66±0.16	0.66±0.12
NREM (min)	452.33±30.51	417.11±55.61	461.42±52.97
NREM Bout Duration (min)	1.37±0.16	0.91±0.24	1.22±0.37
REM (min)	39.09±10.97	30.07±10.75	26.13±6.45
REM Bout Duration (min)	0.63±0.15	0.42±0.19	0.52±0.17

Lights off			
Wake (min)	444.00±42.74	467.26±54.82	308.77±47.7^b,f^
Wake Bout Duration (min)	2.22±0.94	2.61±1.59	1.08±0.44^a,e^
NREM (min)	259.41±43.94	238.1±54	385.92±42.96^c,f^
NREM Bout Duration (min)	1.17±0.23	1.08±0.42	1.19±0.31
REM (min)	16.59±6.63	14.65±4.98	25.32±6.46^a,d^
REM Bout Duration (min)	0.67±0.28	0.46±0.25	0.59±0.13

Averages± standard deviations shown. Values are given for the average plus/minus standard deviation. There were significant interactions between genotype and time of day for amounts of wake (p = 0.0002), NREM sleep (p = 0.001) and REM sleep (p = 0.002). ^a^significantly different from wildtype p<0.05, p>0.01; ^b^significantly different from wildtype p<0.001; ^c^significantly different from wildtype p<0.0001; ^d^significantly different from Homer1a heterozygote p<0.05, p>0.01; ^e^significantly different from Homer1a heterozygote p<0.001; ^f^significantly different from Homer1a heterozygote p<0.0001.

The Homer1a KO mice have less waking than either the wildtype mice or the heterozygotes, but again only during the active dark period (Figure 3b; Table 2). There are no significant differences in wakefulness between the genotypes during the lights on period. Data for 12×2 hour intervals across the day in the wildtype, heterozygote and Homer1a KO are shown in Table S1 (NREM sleep), Table S2 (REM sleep) and Table S3 (wake). The reductions in wakefulness in the Homer1a KO are confined to the lights off period.

### Homer1a knockout mice are unable to sustain long bouts of wakefulness

**Figure 4:**
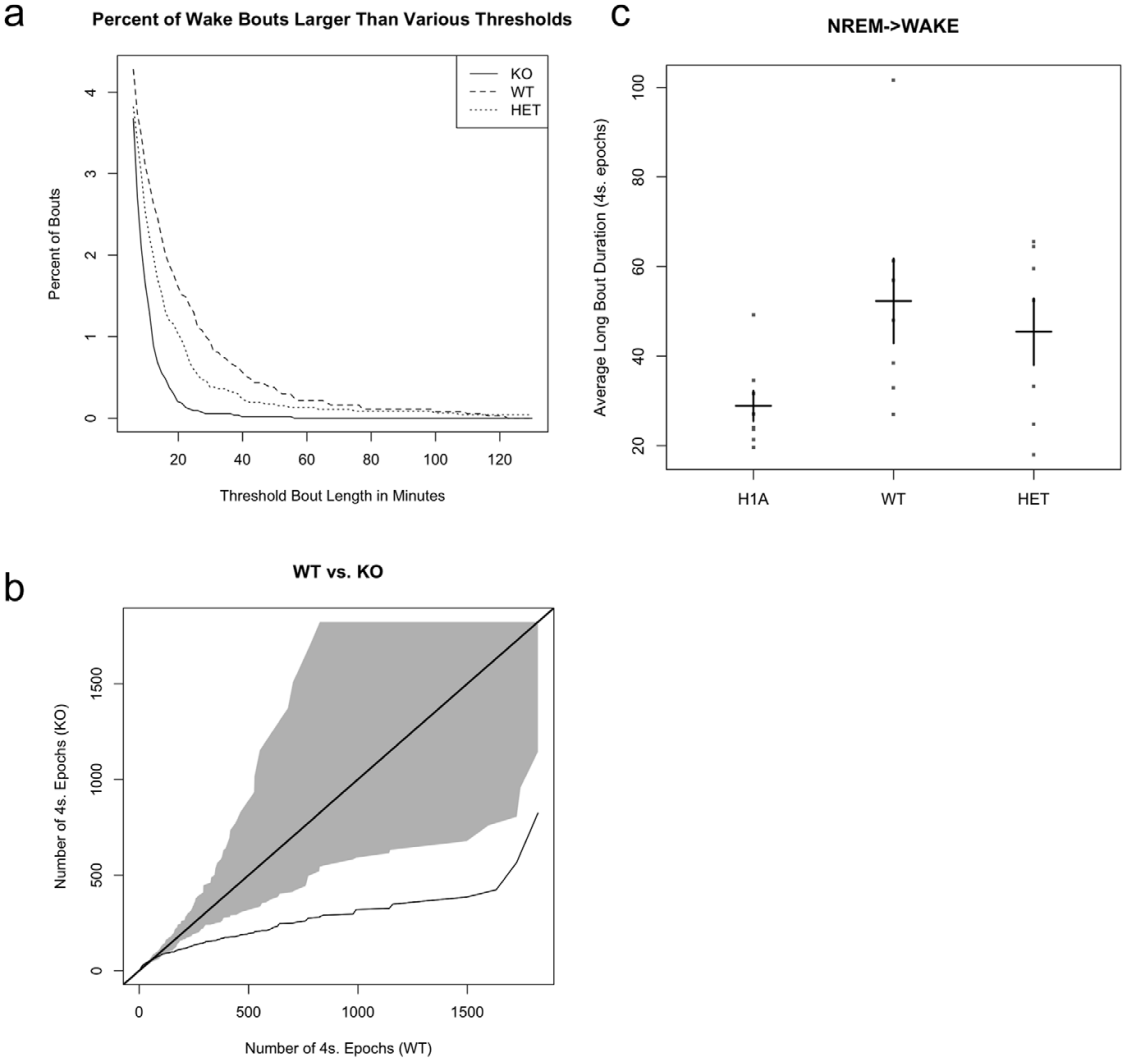
Loss of Homer1a reduces the ability to sustain long wake bouts. A) Percent of wake bouts larger than various thresholds for Homer 1a knockout (KO), wildtype (WT) and heterozygote (HET). The plot shows that for any given bout length threshold, WT and HET have a larger fraction of wake bouts that meet or exceed that threshold as compared to H1A. The number of bouts on the Y axis is small since most bouts are short, whereas the long bouts make up more of the actual duration of wakefulness. B) Q-Q plot for WAKE bout durations given as number of 4-second epochs for Homer1a knockout (KO) (Y axis) and wildtype (WT) mice (X axis). Identical distributions would fall on the line of identify; the grey area on the plot is the estimated variance around the line of identity (null region). The plot show that the Q-Q line falls outside of the grey null region indicating that the bout durations distributions for KO and WT are statistically different from one another. In particular, WT mice have much longer bouts of wakefulness than KO. C) Plot showing that the distribution of the average bout duration slab size in the Homer1a mutant, WT and heterozygote during NREM to wake period. The average slab (composed of long bouts) size is reduced in the Homer1a KO.

Examination of the sleep bout duration indicates that there were no significant differences amongst the three genotypes. However, analysis of wake duration revealed an overall effect of genotype (p = 0.037). Consistent with the reduced wakefulness during the lights off period, the average wake bout during the dark active period in the Homer1a KO mice is significantly shorter than in either the wildtype or the heterozygote (Figure 3c). There are no significant differences in sleep or wake bout duration between the genotypes during the lights on period. The difference in wake bout duration is due to the inability of the Homer1a KO mice to sustain long bouts of wakefulness. In Figure 4a, we examined wake bout duration by strain for various threshold bout lengths. In particular, for a given threshold and strain, we counted the number of wake bouts that met or exceeded that threshold and then divided that by the number of overall wake bouts. We allowed the threshold to vary to generate this plot. As can be seen, regardless of the threshold, wildtype and heterozygote have a greater percentage of wake bouts that exceed that threshold as compared to H1a. Indeed, the phenomenon evidenced in the plot also holds across individual mice. For instance, the longest wake bout experienced by any H1a mouse is 54 minutes and 50 seconds (823 epochs). However, all wildtype and heterozygote mice experience bouts longer than this.

A more formal test of the hypothesis that wake bouts for H1a differ from those of wildtype and heterozygote is given by QQ probability plots as shown for WT and H1a in Figure 4b (for a more complete description and explanation of QQ plots please see the Methods section and Figure S2). Homer1a KO mice do not have long bouts of wakefulness. The wildtype mice have much longer right tails in their wake bout duration distributions compared to the knockout. Wildtype and heterozygote mice have comparable distributions.

In mice the distribution of sleep and wake bouts is not normal; there is a mixture of short and long bouts of each state [21]. We recently described a new statistical model to allow us to obtain parameters to describe this mixture distribution [22] that we have applied to the data from wildtype, Homer 1a heterozygote and Homer 1a knockouts. This model takes into account both the “spike and slab” nature of the distribution of bouts in each state where the spike represents the short bouts and the long bouts are captured by the slab. This new analysis indicates that it is the average duration of the long wake bouts (slab) that are more reduced in the H1a KO mice (Figure 4c).

### Homer1a knockout mice have a normal homeostatic EEG response

There is no significant difference in the homeostatic response to sleep deprivation as measured by the increase in delta power following 6 hours of sleep deprivation between Homer1a KO, Homer1a heterozygote and wildtype controls (Figure S3; Table S4). There were also no significant differences in the time constant of decline of delta power during recovery sleep following sleep deprivation between the three genotypes (Figure S2; Table S4).

### The circadian period is shortened in the Homer1a knockout

Examination of the circadian phenotype by wheel running indicates that the Homer1a KO mice, like the wildtype littermate controls, displayed a circadian rhythm during constant dark conditions (Figure S3). A 1-way ANOVA comparing the circadian period between Homer1a KO mice, the heterozygote and wildtype littermates indicates that there is a main effect of genotype (Welch F test, F = 37.84, p = 1.1×10−7). The Homer1a mice had a shorter period with the average period length being 22.89±0.47 hr compared to the wildtype mice with a tau of 23.35±0.28 hr (p = 0.01) and to the Homer1a heterozygotes that have a tau of 23.89±0.13 (p = 0.0001). There was no significant difference in the circadian period between wildtype and heterozygote mice.

### CREB hypomorph mice have changes in wakefulness similar to Homer1a KO mice and fail to upregulate Homer1a with sleep deprivation

Mice with very low levels of CREB due to deletion of the alpha and delta isoforms of CREB also have reduced wakefulness during the early part of their nighttime active period [23], i.e., similar to what we report here in Homer1a KO mice. Homer1a is a gene whose expression is affected by CREB [24]. To examine whether the reduction in wakefulness in CREB hypomorph mice may be mediated, at least in part, by reduced increases in Homer1a with wakefulness we sleep deprived CREB αΔ hypomorph mice and their wildtype littermates for 1 hour and carried out Q-PCR on their cerebral cortices. CREB hypomorphs that had been sleep deprived for 1 hour showed no increase in expression of homer1a transcript with sleep deprivation whereas their wildtype littermates subjected to the same duration of sleep deprivation showed a robust six-fold increase (p<0.000001; Figure S3).

## Discussion

This study indicates that Homer proteins play a role in sustaining sleep/wake behavioral states in Drosophila and mice that are distinct from the predictions of the “flip-flop” model. In Drosophila, lack of Homer leads to alteration in the ability to sustain both sleep and wakefulness but the effect on sleep consolidation is greater. The Homer null flies have no alteration in clock period but do have an altered response to sleep deprivation. Their initial response to sleep deprivation is similar to wildtype but they show recovery sleep over much longer time periods, i.e., their recovery sleep does not efficiently dissipate the drive for sleep. This may, in part, be related to their sleep being fragmented with short bouts, even with increased drive for sleep. The requirement for Homer in Drosophila to sustain sleep is so marked that even during recovery sleep there is no increase in sleep bout duration after 4 and 6 hours of sleep deprivation as normally occurs, and sleep is solely increased by an increased number of sleep bouts.

In mice the major difference in the sleep/wake phenotype of the Homer1a knockout is in their ability to sustain long bouts of wakefulness. In mice, wakefulness exists in two different states, i.e., short transient bouts of wakefulness and long bouts of sustained wakefulness that occur in the early part of the lights off period [21], [25]. The different durations of bouts of sleep and wakefulness have been analyzed using survival curve analysis [26], [27], [28], [29], [30], [31], [32], [33]. An alternative approach to characterize the distribution of bout lengths is a mixture distribution with a spike (short bouts) and slab (long bouts in the tail of the distribution) [22]. Analysis using this approach reveals that the largest differences between in-bred strains in both sleep and wakefulness is in the duration of the long bouts [22]. Our analysis of the distribution of bout lengths of wakefulness reveals that Homer1a markedly influences the duration of long bouts of wakefulness that a mouse can sustain. It is likely that the molecular mechanism by which this occurs involves Homer1a interactions with the group I metabotropic glutamate receptors; this will be evaluated in future studies.

The Homer1a sleep phenotype is unique and different than phenotypes that are commonly associated with sleep disruption. Most of the other studies have focused on sleep homeostasis after deprivation or transition between sleep and wake. The orexin KO mice display reduced waking and increased NREM and REM across the lights on period [34]. There is wake and NREM fragmentation in both light and dark with transitions from wake to REM sleep. This occurs rarely in wildtype mice. The histidine decarboxylase KO mice display reduced sleep and wake with fragmentation with a reduction in the mean wake episode duration over 24 hr to 3.7 min±0.1 instead of 4.6 min±0.2 seen in wildtype mice [35]. In contrast the Homer1a KO has average wake bout duration of 0.8±0.2 over 24 hours, i.e., a much greater reduction in wake bout duration. The data on the Dopamine [beta] -hydroxylase knockout mice which are deficient in noradrenaline are mixed. One study find that these mice display decreased waking across the day and night and increased sleep (both NREM and REM) with reduced wake and REM bout duration over 24 hours while an earlier study finds no sleep-wake differences between the knockout and wildtype mice [36]. Our data also reveal a strong time of day effect of the Homer1a knockout in mice. This time-of-day effect has also been described for other mutants, e.g., CREB hypomorph [23], and histidine decarboxylase knockout [35]. The basis for this time-of-day effect is currently unknown.

Our data imply that the traditional wake-active neuronal systems such as noradrenaline cells in the locus coeruleus [2], histamine cells in the tuberomammillary nucleus [3], dopamine cells in the periaquaductal gray [4] and orexin cells in the lateral hypothalamus [5] are not sufficient on their own to produce long bouts of wakefulness. Moreover, the same can be said of the recently described glutaminergic pathway for arousal originating in the pons [37]. Our data suggest that there is a mechanism that is mediated by Homer1a that is required for sustained bouts. Thus, it is conceivable that these wake-active systems are sufficient to wake animals up as is shown, for example, by optogenetic activation of orexin cells [6], but not to produce sustained bouts of wakefulness. Our observations add further support to the arguments [7] that sleep and wakefulness are not simply determined by a flip-flop between wake-active and sleep-active cells [1], although previous work challenging this concept did not account for the recently described glutaminergic arousal pathway [37].

Differences in the Homer1a KO compared to wildtype or heterozygotes during the lights on and not the lights off period are not because of absence of a functioning circadian clock. Studies involving changing animals from light/dark to dark/dark conditions reveal a robust circadian rhythm in the Homer1a knockouts. Thus, they have a functioning clock. However, the period of the circadian rhythm is significantly less in the knockout compared to the wildtype or heterozygote. It has been demonstrated that light induces Homer-1a expression in rat SCN both in the early and late night [38] and it remains to be investigated whether this is true in mice as well.

We did not find an effect of loss of Homer1a in the response to sleep deprivation as measured by increase in delta power following 6 hours of sleep deprivation or in decline of delta power during recovery sleep. While Homer1a has been proposed as a strong candidate to explain the quantitative trait locus for sleep homeostasis in mice [15], [17] it is not the only candidate. Other candidates explaining the QTL are Ntrk2 which encodes a receptor that binds BDNF, transmembrane protein 161B, arylsulfatase B, chondroitin sulfate proteogycan 2, mutS homolog 3 of *E. coli* and predicted gene EG667132 [17]. Expression of the *bdnf* gene is known to parallel delta power; it increases with wakefulness and decreases with recovery sleep [39].

The signaling pathways involved in Homer1a induction include the MAP kinase cascade [40]. Studies of cultured striatal neurons indicate that the increased expression of Homer1a produced by dopamine is mediated by CREB [24]. The promoter region of the Homer 1 gene contains multiple CRE sites [41]. Mice with very low levels of CREB due to deletion of the alpha and delta isoforms of CREB also have reduced wakefulness during the nighttime active period [23], i.e., similar to what we report here in Homer1a knockout mice. We found that short durations of extended wakefulness, i.e., 1 hour, that lead to increased expression of Homer1a in cortex of wildtype mice do not do so in CREB hypomorph mice, indicating that the reduction in wakefulness in CREB hypomorph mice may be mediated by reduced Homer1a during wakefulness. Thus, CREB may promote wakefulness by increasing expression of Homer1a.

In conclusion, our data support the notion that Homer scaffolding proteins are required for maintenance of behavioral state and that consolidation of sleep and wake is governed by molecules other than traditionally known neurotransmitters. We note that in a model of competitive actions of H1a versus cross-linking forms of Homer, that up-regulation of H1a is functionally equivalent to down regulation of cross-linking Homer. Thus, despite evolutionary changes in Homer gene structure and copy number, Drosophila and mice share the functional consequence of reduced Homer cross-linking during wakefulness and increased cross-linking during sleep. The cellular substrate of this effect, and whether it is mediated by ascending pathways or targets of these pathways in higher brain areas, will be critical to evaluate in the future.

## Materials and Methods

### Flies

*Drosophila melanogaster* adult females of homer^R102^ and the background wild type strain Canton-S [w CS(10)] were collected within 24 hours after eclosion, placed in locomotor activity tubes and were maintained at 25°C in a 12∶12 light: dark cycle on 5% sucrose 1% agar.

### Deficiency studies

To map the altered phenotype to Homer, the deficiency lines Df(2L)ED7007 and Df(2L)ED6569 that are in the genome region of Homer were crossed with Homer^R102^ to create homer^R102^/Df(2L)ED7007 and homer^R102^/Df(2L)ED6569 hemizygotes. CS flies were also crossed with the same deficiency lines.

### Mice

All experiments were performed on male mice, 10 wk of age±1 wk, maintained on 12 light/dark cycle (lights on 0700; 80 Lux at the floor of the cage) in a sound attenuated recording room, temperature 22–24°C. Food and water were available ad libitum. Animals were acclimated to these conditions for 10–14 days before beginning any studies. All animal experiments were performed in accordance with the guidelines published in the NIH Guide for the Care and Use of Laboratory Animals and were approved by the University of Pennsylvania Animal Care and Use Committee.

### Homer mutant mice

The Homer1a null mice were generated as described by [20]. Briefly, exons 6–10 of Homer1c were fused with exon 5 so that the H1a splice form would not exist and the Homer1b, 1c and 1d would not altered. There is no change of H1b/c level in the H1a KO mice (see supplemental figure****S2F in Hu et al, 2010 [20]). These mice which are in a C57BL/6J background were crossed with C57BL/6J mice to generate Homer1a heterozygotes. The heterozygous Homer1a mice were then crossed with each other to generate wildtype littermate controls, heterozygous Homer1a and Homer1a KO mice.

### CREB mutant mice

CREB mutant αΔ hypomorph mice were generated as described by Graves et al [23]. Genotyping was performed by PCR as described [42].

### Genotyping

Mice were genotyped using the REDExtract-N-Amp Tissue PCR kit (Sigma) and standard PCR techniques. The primers for genotyping the Homer1a knockout, heterozygote and wildtype mice were as follows:

H1aB2: 5′-AGTCAAAGAGGTCCCTCTGTTCTTG-3′ (reverse)

H1aB3: 5′-TCATGTTTACAGTCCAGTAATGCC-3′ (reverse)

H1aA3: 5′-TGTGACACAGAACTCAGCCAAG-3′ (forward)

The primers used to genotype the CREB mutant mice were as follows:

CREB alpha/delta 5′ primer: CAGGGACCATTCCTCATTTCCT

CREB alpha/delta 3′ primer: GCTGGGCTTGAACTGTCATTTG

CREB alpha/delta NEO primer: GGACAGGTCGGTCTTGACAAAA

### Sleep-Wake Behavioral Monitoring

#### Flies

Rest/activity was recorded using beam breaks in the Drosophila Activity Monitoring System (DAMS) (Triknetics, Waltham, MA) as described in Naidoo et al, 2007 [43] or by video as described previously [18]. Sleep was defined as 5 minutes or more of continuous inactivity for both methods based on accepted criteria [19], [44], [45]. Total minutes of sleep and wake were calculated in 12 hour day or night periods and over the 24 period. Numbers of sleep and wake bouts and their duration were also determined.

#### Sleep/Wake Architecture Analysis

The DAMS and video data were analyzed using custom software [18] to obtain the following sleep and wake parameters: total sleep; total wake; average sleep bout duration; number of sleep bouts, average wake bout duration and number of wake bouts.

#### Sleep deprivation (SD)

We used video analysis to record two days of baseline behavior and then sleep deprived Homer^R102^ and CS flies for 4 or 6 hours by tapping during the dark phase when the fly was seen to be quiescent. We chose to end all deprivations at ZT 22 because the background CS strain displays the most consolidated rest between ZT16–22. Time matched controls were maintained in the monitors without intervention. After SD, the flies were allowed to recover for 24 hr during which their behavior was recorded by video. Recovery sleep was calculated by subtracting the total sleep per hour on baseline day from total sleep per hour post sleep deprivation.

#### Estimation of Circadian Period

For analysis of circadian behavior, activity counts were collected in 1-minute bins in DD over a 6-day period and analyzed using ClockLab (Actimetrics).

#### Mice – EEG/EMG Recording of Sleep and Scoring of Sleep/Wake and Substages of Sleep

Sleep-wake behavior was recorded via EEG/EMG as described in [46]. Wake, NREM and REM sleep were manually scored as described previously [46] in 4-second epochs during 24 hour baseline recordings.

#### Sleep Deprivation

Mice were sleep deprived for 6 hours. Deprivation was initiated at lights-on (7:00 AM), and performed through gentle handling [46], following an acclimatization period for handling procedures. To address the response to sleep deprivation, we measured the increase in delta power in the EEG during recovery NREM sleep following 6 hours of sleep deprivation and analyzed delta power in the frequency range 1–4 Hz in 4-second epochs of NREM sleep using the Somnologica software system (Medcare). Epochs with artifacts were excluded from analysis. Following Franken et al [25], we defined the baseline of delta power as the average over the last 4 hours of the lights on period (3 pm–7 pm) on the day preceding the sleep deprivation. The homeostatic response was then assessed by calculating the delta power averaged over the first 225×4-second epochs of NREM sleep in the initial part of recovery sleep following 6 hours of deprivation. For each mouse we defined the homeostatic response as the increase in delta power in initial recovery sleep compared to baseline delta power and expressed this as a percentage increase.

To assess the exponential decline in delta power during recovery sleep following sleep deprivation, we calculated the average delta power for consecutive 225×4-second (15 minutes) epochs of NREM sleep between 1 pm and 7 pm. Since there could be other stages (wake, REM sleep) as time progressed, it took more than 15 minutes to accrue the 225×4-second epochs of NREM. We assessed the time for each such measure as the temporal mid-point of the 225 epochs. We examined for each mouse the relationship between the log of delta power and time and specifically computed the linear slope of this relationship as a measure of the time constant of the decline in delta power.

#### Circadian Period

Circadian period was assessed as described [47]. Raster plots of activity and the length of each circadian day (τ) were assessed using Actimetrics Clocklab software. The period of the clock for each mouse is defined by the degree of phase advance of the amount of wheel running in constant darkness.

### Biochemical and Molecular Biology Methodology

The Drosophila Homer antibody, which was made against full length Homer, recognizes a 47 kilodalton protein from adult fly heads by western blotting. Lysates from Homer^R102^ fly heads probed with this antibody did not produce a signal indicating that the mutant flies do not make full length Homer (Figure S1).

#### Western Blotting

Protein expression of Homer in single fly heads was determined by Western analysis using the protocol described in [43]. Blots were incubated with rat polyclonal antibody against Drosophila Homer (1∶1000, gift from Ulrich Thomas). After incubation with horseradish peroxidase-conjugated secondary antibody (anti-rat 1∶3000, Sigma), protein bands were detected and analyzed by enhanced chemiluminescence (Pierce Supersignal) and quantitative imaging (AlphaInnotech Fluorochem 8900). Densitometry was performed using the Alphaease FC software. Alternatively, IR conjugated secondary antibodies were used and protein bands were detected and quantified by infra red imaging on an Odyssey (LiCor).

#### Quantitative PCR

Quantitative PCR was carried out on the cerebral cortices of CREB mutant and wildtype littermate mice that had been sleep deprived for 1 hour and in spontaneously sleeping mice of the same genotypes. The sample of cerebral cortex was composed of the M1 and M2 areas and included the midline located Cg1 and Cg2 regions. Total RNA was extracted using TRIzol (Invitrogen) and further cleaned using the RNAeasy purification kit (Qiagen).

The relative transcript level was established by the “ΔΔ method” as described previously [48]. The Apc gene was used as an internal standard; this gene was identified as unchanged in its expression during sleep or sleep deprivation in the cerebral cortex or hypothalamus of C57BL/6 mice [16]. A second standard reference consisting of a pool of all the samples was used to generate a standard curve. Expression levels of the Homer1a transcript were determined based on this standard curve. The primers for the *Homer1a* gene were designed from a region that includes intron 5, which contributes a nucleotide sequence found in *Homer1a* but not *Homer1c*.

The following primers and probe were used for *Homer1a*:

5′-end primer-5′TGTCCTGCTGTAAATAAAGCCATCT-3′;

3′-end primer-5′-AGTATAATCATCTTAACTATCTAGCACCTTCTG-3′. The probe for *Homer1a* contained the 6-FAM at the 5′-end and the Black Hole Quencher (3BHQ_1) at the 3′-end, and had the following nucleotide sequence: 5′-6-FAM-AGCAGCGCCTTGAGTTCTTGTGGCTA-3BHQ_1–3′. The final PCR was performed with Homer1a-specific primers: upper strand primer: 5′-AACACTGTTTATGGACTGGG-3′, lower strand primer: 5′-TGATTGCTGAATTGAATGTGTACCTATGTG-3′. The custom made primers and probes were used at a concentration of 50 μM and 20 μM respectively.

### Statistical Analyses

#### Flies

A mixed model analysis of variance (ANOVA) was used to estimate least squares mean contrasts of interest between genotypes, overall, and at specific times-of-day.

#### Amounts of Sleep and Wake in Mice

The analysis for comparisons of data from the 24-hour recordings used a mixed model ANOVA, with a factor for genotype (wildtype, heterozygote and Homer1a KO) and a repeated measures factor of time period. Separate models operationalized time into: (1) an overall 24 hour time period; (2) 2 twelve-hour time periods (light/dark); and (3) 12 two-hour time periods. A logarithm variance stabilizing transformation was used where indicated by the Shapiro-Wilk statistic and inspection of normal probability plots.

#### Distribution of Wake Bout Duration

We compared the distributions of WAKE bout durations of the three strains using Q-Q plots. Q-Q plots are a commonly used graphical method to compare two probability distributions. They compare two distributions by plotting their empirical quantiles (i.e., the sorted values) against one another. When the distributions are the same, the Q-Q line should lie approximately on the *y* = *x* line (Figure S2). We performed this analysis for distributions of bout durations of wakefulness comparing Homer 1a knockout to both wildtype and heterozygote mice.

In addition a novel statistical method that takes into account both the “spike and slab” nature of the distribution of bouts in each state and the fact of that the duration of each bout is dependent on the previous state was used as previously described [22]. In this spike and slab model the spike represents the short bouts and the slab represents the long bouts.

#### Sleep Deprivation Response

For the sleep deprivation response, we assessed differences between genotypes in our two measures, i.e., magnitude of sleep homeostatic response and time constant of decline of delta power during recovery sleep using a one-way ANOVA with the main factor being the 3 genotypes. The analysis of delta power changes was identical to that described by Franken et al [25].

#### Circadian Period

For comparison of circadian period, we used a one-way ANOVA with the main factor being genotype (three types–wildtype, heterozygotes and homozygote KO mice). Pair-wise comparisons between genotypes were done.

## Supporting Information

Figure S1 A) Homer^R102^ flies do not make Homer protein. Representative immunoblot showing the absence of D-Homer in the homer^R102^ flies as detected by an antibody made to full length D-Homer and present in the Canton-S (CS) background strain. Each lane represents a single fly head. B) Representative western blot showing homer expression in individual fly heads following 6 hr sleep deprivation (SD) comparing the three lanes from control flies to 3 from sleep deprived (SD) flies. C) Densitometric quantification of homer expression in female heads (n = 9) following 6 hr SD (▪). The density of each band was normalized to that of the control. Data shown are mean and standard deviation. The mean ratio of Homer protein from sleep deprived flies to controls was 0.62 (95% CI was 0.44 to 0.99). The non-parametric analog to the t-test was also significant at p = 0.039 (Wilcoxon signed rank test). D) The Homer^R102^/Df and CS/Df hemizygotes display very different sleep phenotypes. There are significant differences in sleep bout number (p≤0.001) and sleep bout length (p≤0.001) between Homer^R102^/Df and CS, CS/Df and Homer^R102^/CS. There are no significant differences between Homer/Df and Homer/Homer. Shown are the average percent time sleeping, number of sleep bouts and mean sleep bout length in minutes with standard deviations for Homer^R102^/ Homer^R102^ (n = 20); Homer^R102^/Df (n = 30); CS (n = 16); CS/Df (n = 13) and Homer^R102^/CS (n = 13).
(PDF)

Figure S2 Representative Q-Q plot illustrating the comparison of two probability distributions. The black line falling within the grey lines indicates that the null hypothesis is retained (left panel). The black line outside the grey region rejects the null hypothesis of equality of distribution (right panel).
(PDF)

Figure S3 A) Homeostatic response to 6 hours of sleep deprivation measured as a percent of baseline from one mouse of each genotype (WT, HET, KO). The representative line graphs illustrate the decline in delta power in the 3 strains over 15 consecutive epochs. There was no significant difference in the decline of delta power during recovery sleep. B) Representative activity records of Homer 1a knockout, heterozygote and wildtype mice entrained to an LD 12∶12 cycle and subsequently placed in constant darkness (DD) on day 10 as indicated by arrows. Successive days are plotted from top to bottom, and *x*-axis represents a double plotted 48-h period of activity. Black bar on top indicates dark phase of the LD cycle. The Homer 1a knockout has a reduced circadian period. C) Expression levels of Homer 1a transcripts in the cerebral cortex of CREB αΔ mutant mice (Mut) and wildtype littermates (WT) after 1 hour of total sleep deprivation (SD) (▪) and spontaneous sleep (SS) (□); mean and standard deviation shown. 1 hour SS (n = 4/group); 1 hour SD (n = 3/group). Wildtype mice display a significant increase in Homer 1a transcript after 1 hour of sleep deprivation compared to the mutant mice. The mean expression level of Homer1a mRNA in the CREB αΔ hypomorph is 50.04±26.66 compared to the wildtype which has 301.98±45.72 (p = 2.6 e-9; TTEST; n = 3). The wildtype mice also express significantly more Homer 1a transcript than mice sleeping spontaneously over 1 h (88.4±5.6; p = 2.7e-11; TTEST).
(PDF)

Table S1 Average amount of NREM and NREM bout duration for Homer 1a wildtype, heterozygote and knockout mice measured in 2 hour time periods. Values are given for the average plus/minus standard deviation. Values significantly different from Homer1a knockout denoted by a = p<0.05 p>0.01; b = p<0.01 p>0.001; c = p<0.001 p>0.0001; d = p≤0.0001.
(PDF)

Table S2 Average amount of REM and REM bout duration for Homer 1a wildtype, heterozygote and knockout mice measured in 2 hour time periods. Values are given for the average plus/minus standard deviation. Values significantly different from Homer1a knockout denoted by a = p<0.05 p>0.01.
(PDF)

Table S3 Minutes of waking and wake bout duration in the 3 genotypes during the lights on and lights off periods in 2 hour intervals. Values are given for the average plus/minus standard deviation. Values significantly different from Homer1a knockout denoted by a = p<0.05 p>0.01; b = p<0.01 p>0.001; c = p<0.001 p>0.0001; d = p≤0.0001
(PDF)

Table S4 Changes in NREM delta determined in 15 minute intervals following 6 hours of sleep deprivation. Delta is measured as % of baseline (100%). Midpoint is the chronological midpoint time of each interval measured from the end of the sleep deprivation period. There are no significant differences between genotypes.
(PDF)
