# Supplementary figures and images for: Role of Homer Proteins in the Maintenance of Sleep-Wake States
Source: PLoS One. 2012 Apr 20;7(4):e35174. doi: 10.1371/journal.pone.0035174 (PMC3332115; doi:10.1371/journal.pone.0035174)

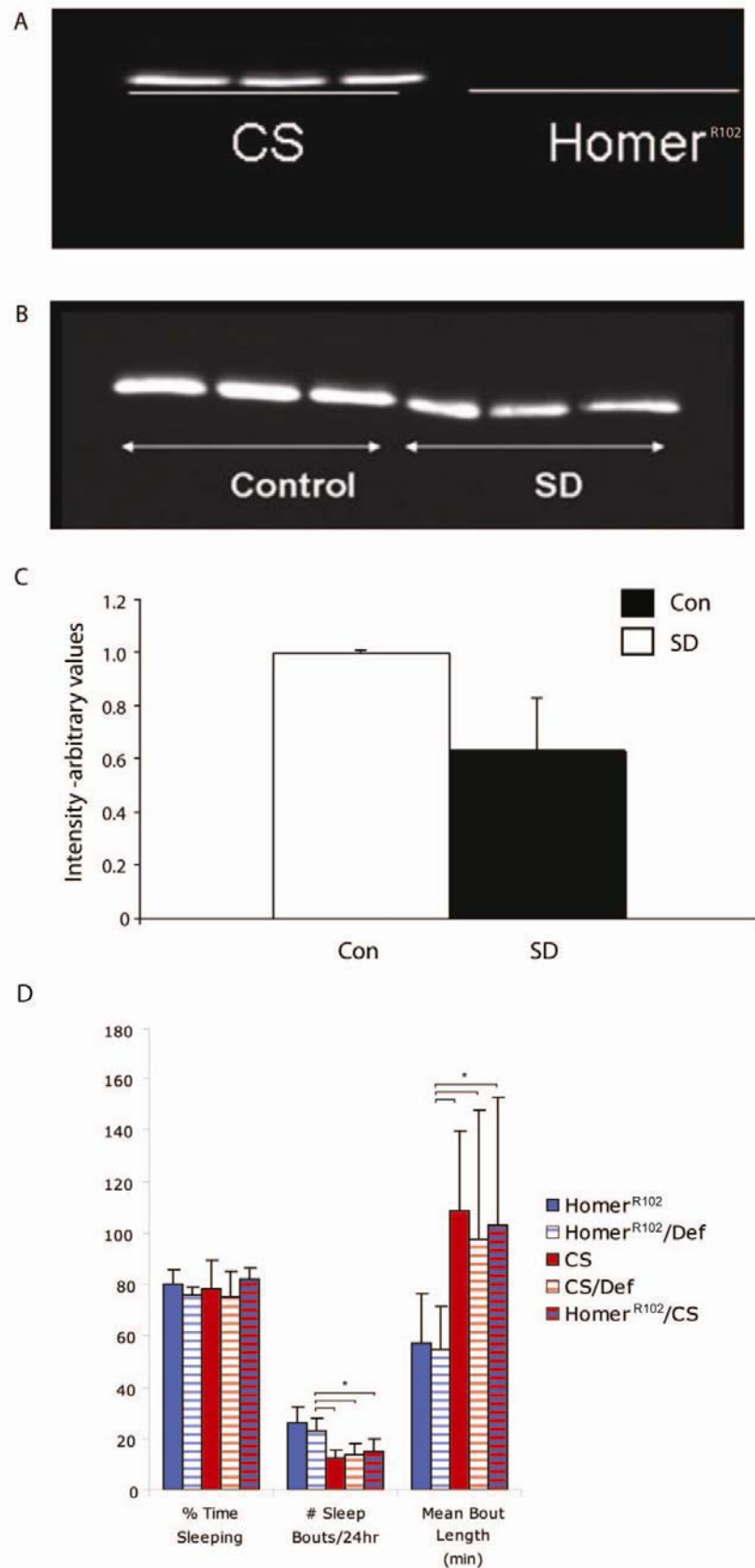

FIGURE S1

Supplement: Figure S1 — A) HomerR102 flies do not make Homer protein. Representative immunoblot showing the absence of D-Homer in the homerR102 flies as detected by an antibody made to full length D-Homer and present in the Canton-S (CS) background strain. Each lane represents a single fly head. B) Representative western blot showing homer expression in individual fly heads following 6 hr sleep deprivation (SD) comparing the three lanes from control flies to 3 from sleep deprived (SD) flies. C) Densitometric quantification of homer expression in female heads (n = 9) following 6 hr SD (▪). The density of each band was normalized to that of the control. Data shown are mean and standard deviation. The mean ratio of Homer protein from sleep deprived flies to controls was 0.62 (95% CI was 0.44 to 0.99). The non-parametric analog to the t-test was also significant at p = 0.039 (Wilcoxon signed rank test). D) The HomerR102/Df and CS/Df hemizygotes display very different sleep phenotypes. There are significant differences in sleep bout number (p≤0.001) and sleep bout length (p≤0.001) between HomerR102/Df and CS, CS/Df and HomerR102/CS. There are no significant differences between Homer/Df and Homer/Homer. Shown are the average percent time sleeping, number of sleep bouts and mean sleep bout length in minutes with standard deviations for HomerR102/ HomerR102 (n = 20); HomerR102/Df (n = 30); CS (n = 16); CS/Df (n = 13) and HomerR102/CS (n = 13). (PDF) [file pone.0035174.s001.pdf]

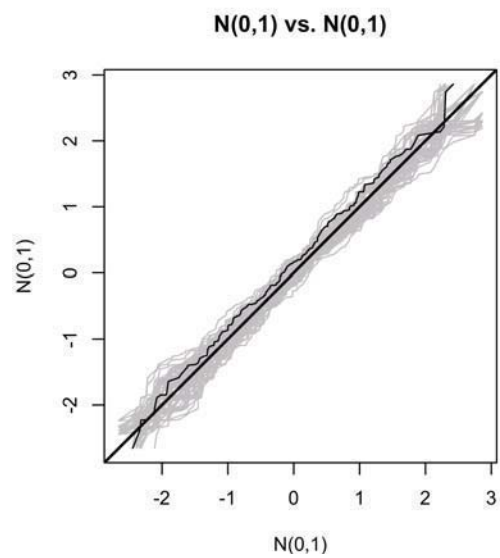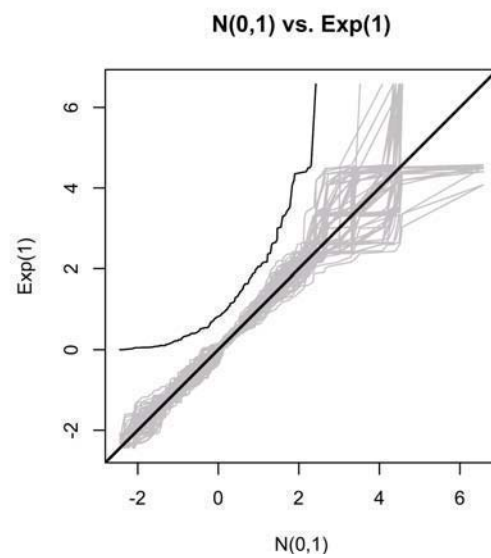

FIGURE S2

Supplement: Figure S2 — Representative Q-Q plot illustrating the comparison of two probability distributions. The black line falling within the grey lines indicates that the null hypothesis is retained (left panel). The black line outside the grey region rejects the null hypothesis of equality of distribution (right panel). (PDF) [file pone.0035174.s002.pdf]

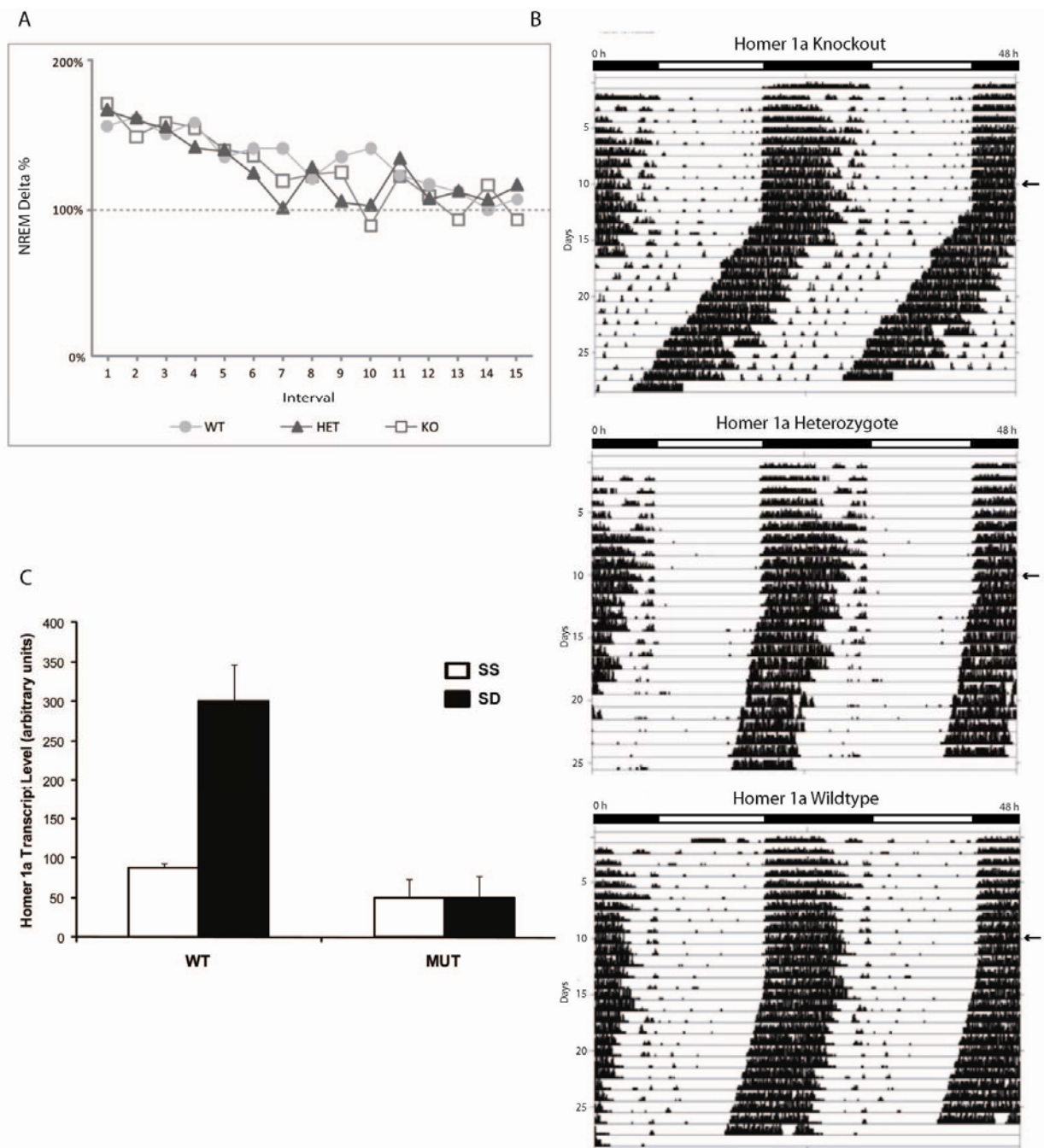

FIGURE S3

Supplement: Figure S3 — A) Homeostatic response to 6 hours of sleep deprivation measured as a percent of baseline from one mouse of each genotype (WT, HET, KO). The representative line graphs illustrate the decline in delta power in the 3 strains over 15 consecutive epochs. There was no significant difference in the decline of delta power during recovery sleep. B) Representative activity records of Homer 1a knockout, heterozygote and wildtype mice entrained to an LD 12∶12 cycle and subsequently placed in constant darkness (DD) on day 10 as indicated by arrows. Successive days are plotted from top to bottom, and x-axis represents a double plotted 48-h period of activity. Black bar on top indicates dark phase of the LD cycle. The Homer 1a knockout has a reduced circadian period. C) Expression levels of Homer 1a transcripts in the cerebral cortex of CREB αΔ mutant mice (Mut) and wildtype littermates (WT) after 1 hour of total sleep deprivation (SD) (▪) and spontaneous sleep (SS) (□); mean and standard deviation shown. 1 hour SS (n = 4/group); 1 hour SD (n = 3/group). Wildtype mice display a significant increase in Homer 1a transcript after 1 hour of sleep deprivation compared to the mutant mice. The mean expression level of Homer1a mRNA in the CREB αΔ hypomorph is 50.04±26.66 compared to the wildtype which has 301.98±45.72 (p = 2.6 e-9; TTEST; n = 3). The wildtype mice also express significantly more Homer 1a transcript than mice sleeping spontaneously over 1 h (88.4±5.6; p = 2.7e-11; TTEST). (PDF) [file pone.0035174.s003.pdf]
