# Supplementary material for: Role of Homer Proteins in the Maintenance of Sleep-Wake States
Source: PLoS One. 2012 Apr 20;7(4):e35174. doi: 10.1371/journal.pone.0035174 (PMC3332115; doi:10.1371/journal.pone.0035174)
Supplement: Table S1 — Average amount of NREM and NREM bout duration for Homer 1a wildtype, heterozygote and knockout mice measured in 2 hour time periods. Values are given for the average plus/minus standard deviation. Values significantly different from Homer1a knockout denoted by a = p<0.05 p>0.01; b = p<0.01 p>0.001; c = p<0.001 p>0.0001; d = p≤0.0001. (PDF) [file pone.0035174.s004.pdf]

Supplemental Materials

Table S1 Average amount of NREM and NREM bout duration for Homer 1a wild-type, heterozygote and knockout mice measured in 2 hour time periods. Values are given for the average plus/minus standard deviation. Values significantly different from Homer1a knockout shown in bold and denoted by a= $p<0.05$ $p>0.01$; b= $p<0.01$ $p>0.001$; c= $p<0.001$ $p>0.0001$; d= $p\leq0.0001$.

	Wild-type (n=7)		Homer 1a Het (n=7)		Homer 1a Knockout (n=8)	
Interval	NREM (min)	NREM Bout Duration (min)	NREM (min)	NREM Bout Duration (min)	NREM (min)	NREM Bout Duration (min)
7am-9am	76.84 ± 10.06	1.57 ± 0.22	74.46 ± 16.82	0.97 ± 0.25	81.49 ± 15.95	1.35 ± 0.76
9am-11am	75.07 ± 14.35	1.37 ± 0.16	67.7 ± 8.55	**0.9 ± 0.26[a]**	79.22 ± 10.85	1.2 ± 0.38
11am-1pm	76.9 ± 10.72	1.38 ± 0.15	66.16 ± 9.8	**0.9 ± 0.27[a]**	80.53 ± 14.66	1.18 ± 0.33
1pm-3pm	81.93 ± 8.90	1.36 ± 0.14	81.44 ± 16.17	0.98 ± 0.38	83.67 ± 11.02	1.24 ± 0.32
3pm-5pm	77.44 ± 7.04	1.41 ± 0.39	**70.49 ± 5.67[a]**	0.93 ± 0.34	75.96 ± 10.04	1.28 ± 0.40
5pm-7pm	64.15 ± 8.60	1.16 ± 0.18	**56.87 ± 11.87[b]**	0.87 ± 0.22	60.55 ± 16.22	1.15 ± 0.41
7pm-9pm	**30.05 ± 16.3[b]**	0.94 ± 0.29	**20.99 ± 21.8[c]**	0.55 ± 0.32	46.82 ± 7.64	1.03 ± 0.39
9pm-11pm	**42.95 ± 16.29[c]**	1.11 ± 0.19	**27.54 ± 25.97[d]**	0.64 ± 0.36	66.62 ± 7.78	1.22 ± 0.30
11pm-1am	**41.64 ± 10.40[c]**	1.27 ± 0.31	**36.6 ± 28.76[d]**	1.16 ± 0.75	61.76 ± 12.07	1.10 ± 0.30
1am-3am	**47.85 ± 22.33[d]**	1.28 ± 0.46	**58.09 ± 24.87[d]**	1.22 ± 0.57	70.21 ± 0.43	1.32 ± 0.37
3am-5am	**53.57 ± 13.39[d]**	1.18 ± 0.3	**55.86 ± 27.46[d]**	1.01 ± 0.29	70.74 ± 13.51	1.33 ± 0.50
5am-7am	**43.35 ± 11.49[b]**	1.13 ± 0.21	**39.02 ± 33.12[b]**	0.95 ± 0.57	69.78 ± 7.63	1.2 ± 0.27
