# Supplementary material for: Role of Homer Proteins in the Maintenance of Sleep-Wake States
Source: PLoS One. 2012 Apr 20;7(4):e35174. doi: 10.1371/journal.pone.0035174 (PMC3332115; doi:10.1371/journal.pone.0035174)
Supplement: Table S2 — Average amount of REM and REM bout duration for Homer 1a wildtype, heterozygote and knockout mice measured in 2 hour time periods. Values are given for the average plus/minus standard deviation. Values significantly different from Homer1a knockout denoted by a = p<0.05 p>0.01. (PDF) [file pone.0035174.s005.pdf]

Table S2. Average amount of REM and REM bout duration for Homer 1a wild-type, heterozygote and knockout mice measured in 2 hour time periods. Values are given for the average plus/minus standard deviation. Values significantly different from Homer1a knockout shown in bold and denoted by a= $p<0.05$ $p>0.01$.

	**Wild-type (n=7)**		**Homer 1a Het (n=7)**		**Homer 1a Knockout (n=8)**	
Interval	REM (min)	REM Bout Duration (min)	REM (min)	REM Bout Duration (min)	REM (min)	REM Bout Duration (min)
7am-9am	6.68 ± 2.35	0.72 ± 0.33	5.24 ± 1.89	0.41 ± 0.14	3.5 ± 1.91	0.5 ± 0.37
9am-11am	6.78 ± 3.16	0.56 ± 0.18	5.04 ± 2.26	0.4 ± 0.17	5.07 ± 1.89	0.67 ± 0.25
11am-1pm	6.89 ± 4.41	0.76 ± 0.31	4.9 ± 2.23	0.36 ± 0.18	5.08 ± 2.33	0.47 ± 0.16
1pm-3pm	7.18 ± 2.15	0.71 ± 0.23	6.45 ± 3.65	0.49 ± 0.24	5.63 ± 2.11	0.58 ± 0.21
3pm-5pm	5.79 ± 3.12	0.7 ± 0.36	5.19 ± 3.4	0.39 ± 0.22	4.10 ± 1.95	0.61 ± 0.31
5pm-7pm	5.77 ± 1.61	0.54 ± 0.14	3.25 ± 0.89	0.61 ± 0.64	2.77 ± 1.99	0.37 ± 0.15
7pm-9pm	0.9 ± 0.99	0.81 ± 0.98	0.73 ± 1.15	0.33 ± 0.12	1.75 ± 1.09	0.44 ± 0.37
9pm-11pm	2.1 ± 1.16	0.46 ± 0.15	1.66 ± 1.92	0.43 ± 0.26	3.78 ± 1.91	0.55 ± 0.18
11pm-1am	2.61 ± 0.9	0.72 ± 0.3	1.93 ± 2.33	0.48 ± 0.26	4.20 ± 1.72	0.65 ± 0.24
1am-3am	3.68 ± 2.3	0.8 ± 0.48	**4.1 ± 3.04**[a]	0.43 ± 0.16	5.34 ± 2.07	0.72 ± 0.17
3am-5am	4.64 ± 3.54	0.77 ± 0.39	**3.66 ± 1.86**[a]	0.69 ± 0.9	6.19 ± 2.18	0.67 ± 0.19
5am-7am	2.61 ± 1.93	0.67 ± 0.43	2.57 ± 2.81	0.65 ± 0.51	4.05 ± 0.87	0.68 ± 0.33
