# Supplementary material for: Role of Homer Proteins in the Maintenance of Sleep-Wake States
Source: PLoS One. 2012 Apr 20;7(4):e35174. doi: 10.1371/journal.pone.0035174 (PMC3332115; doi:10.1371/journal.pone.0035174)
Supplement: Table S3 — Minutes of waking and wake bout duration in the 3 genotypes during the lights on and lights off periods in 2 hour intervals. Values are given for the average plus/minus standard deviation. Values significantly different from Homer1a knockout denoted by a = p<0.05 p>0.01; b = p<0.01 p>0.001; c = p<0.001 p>0.0001; d = p≤0.0001 (PDF) [file pone.0035174.s006.pdf]

Table S3: Minutes of waking and wake bout duration in the 3 genotypes during the lights on and lights off periods in 2 hour intervals. Values are given for the average plus/minus standard deviation. Values significantly different from Homer1a knockout shown in bold and denoted by a= p<0.05 p>0.01; b= p<0.01 p>0.001; c= p<0.001 p>0.0001; d= p≤0.0001.

	**Wild-type (n=7)**		**Homer 1a Het (n=7)**		**Homer 1a Knockout (n=8)**	
Interval	Wake (min)	Wake Bout Duration (min)	Wake (min)	Wake Bout Duration (min)	Wake (min)	Wake Bout Duration (min)
7am-9am	36.49 ±10.47	0.83 ± 0.4	40.31 ± 17.79	0.65 ± 0.4	35.01 ± 15.51	0.59 ± 0.27
9am-11am	38.15 ± 13.92	0.86 ± 0.5	47.26 ± 7.93	0.68 ± 0.16	35.72 ± 11.28	0.61 ± 0.34
11am-1pm	36.21 ± 14.3	0.72 ± 0.33	48.93 ± 9.86	0.81 ± 0.36	34.39 ± 15.49	0.59 ± 0.29
1pm-3pm	30.89 ± 9.11	0.57 ± 0.23	32.11 ± 14.53	0.39 ± 0.13	30.71 ± 11.44	0.50 ± 0.16
3pm-5pm	36.77 ± 9.10	0.73 ± 0.29	**44.32 ± 6.72[a]**	0.65 ± 0.19	39.94 ± 10.88	0.74 ± 0.27
5pm-7pm	50.08 ± 9.40	1.05 ± 0.37	**59.89 ± 11.41[b]**	1.02 ± 0.38	56.68 ± 17.52	1.23 ± 0.57
7pm-9pm	**88.99 ± 16.04[a]**	7.84 ± 13.54	**98.28 ± 22.64[c]**	13.99 ± 15.53	71.43 ± 7.56	1.78 ± 0.89
9pm-11pm	**74.95 ± 17.3[b]**	2.53 ± 1.91	**90.8 ± 27.65[d]**	**36.91 ± 56.93[a]**	49.60 ± 9.33	1.03 ± 0.47
11pm-1am	**75.75 ± 10.83[c]**	2.74 ± 1.43	**81.47 ± 30.43[d]**	**21.4 ± 43.92[b]**	54.04 ± 12.49	1.08 ± 0.53
1am-3am	**68.48 ± 24.19[d]**	4.24 ± 6.73	**57.82 ± 27.61[d]**	1.7 ± 1.61	44.45 ± 12.20	0.97 ± 0.46
3am-5am	**61.79 ± 14.70[d]**	1.63 ± 1.07	**60.49 ± 28.74[d]**	2.48 ± 4.13	43.07 ± 15.24	1.01 ± 0.70
5am-7am	**74.04 ± 12.64[b]**	2.19 ± 0.94	**78.41 ± 35.73[b]**	5.01 ± 5.04	46.18 ± 8.42	0.91 ± 0.43
