# Supplementary material for: Role of Homer Proteins in the Maintenance of Sleep-Wake States
Source: PLoS One. 2012 Apr 20;7(4):e35174. doi: 10.1371/journal.pone.0035174 (PMC3332115; doi:10.1371/journal.pone.0035174)
Supplement: Table S4 — Changes in NREM delta determined in 15 minute intervals following 6 hours of sleep deprivation. Delta is measured as % of baseline (100%). Midpoint is the chronological midpoint time of each interval measured from the end of the sleep deprivation period. There are no significant differences between genotypes. (PDF) [file pone.0035174.s007.pdf]

Table S4: Changes in NREM delta determined in 15 minute intervals following 6 hours of sleep deprivation. Delta is measured as % of baseline (100%). Midpoint is the chronological midpoint time of each interval measured from the end of the sleep deprivation period. There are no significant differences between genotypes.

	Homer 1a Wild-type		Homer 1a Het		Homer 1a Knockout	
Interval	MidPoint (min)	MeanDelta (%)	MidPoint (min)	MeanDelta (%)	MidPoint (min)	MeanDelta (%)
0	Baseline	100.00 ± 0.00	Baseline	100.00 ± 0.00	Baseline	100.00 ± 0.00
1	18.7 ± 9.7	180.38 ± 36.62	13.7 ± 4.9	190.06 ± 29.33	12.5 ± 2.2	175.10 ± 47.21
2	38.7 ± 10.6	173.33 ± 28.34	35.6 ± 9.5	184.12 ± 48.86	33.4 ± 2.2	154.24 ± 35.00
3	57.8 ± 11.9	165.68 ± 33.77	54.3 ± 10.1	161.91 ± 37.25	53.2 ± 5.4	143.57 ± 31.73
4	77.8 ± 13.2	152.36 ± 36.04	74.1 ± 10.9	151.78 ± 32.98	73.6 ± 8.1	129.41 ± 17.42
5	97.3 ± 14.2	147.27 ± 25.39	99.3 ± 12.4	153.38 ± 40.79	94.7 ± 13.4	119.68 ± 10.36
6	116.2 ± 15.3	137.20 ± 26.33	117.5 ± 15.4	140.68 ± 41.07	118.1 ± 18.9	114.58 ± 12.95
7	137.4 ± 14.4	126.66 ± 22.73	141.7 ± 18.9	130.19 ± 35.66	143.5 ± 18.5	107.34 ± 7.80
8	160.5 ± 14.4	126.24 ± 18.76	161.5 ± 19.2	129.69 ± 27.95	166.5 ± 18.1	110.38 ± 14.76
9	188.7 ± 11.6	126.58 ± 15.54	185.3 ± 18.4	124.25 ± 33.61	185.6 ± 18.3	102.62 ± 15.22
10	216.9 ± 11.6	121.63 ± 14.79	211.3 ± 18.7	131.10 ± 33.29	208.2 ± 22.8	98.75 ± 9.35
11	242.4 ± 13.4	114.63 ± 17.56	235.5 ± 21.6	121.10 ± 25.72	232.9 ± 27.6	99.41 ± 15.18
12	267.6 ± 16.3	112.12 ± 15.33	258.9 ± 19.9	117.01 ± 22.08	255.8 ± 28.8	100.03 ± 12.25
13	293.8 ± 14.3	117.94 ± 19.99	280.8 ± 20.5	108.68 ± 20.92	280.2 ± 29.5	96.44 ± 7.77
14	320.7 ± 16.1	113.11 ± 20.72	306.9 ± 27.4	108.40 ± 16.36	305.5 ± 33.8	100.70 ± 9.06
15	353.8 ± 31.4	108.75 ± 16.05	337.3 ± 35.3	112.65 ± 20.83	331.6 ± 40.7	95.05 ± 8.84
